# More Jump More Health: Vertical Jumping Learning of Chinese Children and Health Promotion

**DOI:** 10.3389/fpsyt.2022.885012

**Published:** 2022-05-13

**Authors:** Liu Liu, Luo Xi, Wang Yongshun, Zhu Ziping, Ma Chunyin, Qin Peifu

**Affiliations:** ^1^School of Physical Education, Sichuan University, Chengdu, China; ^2^School of Physical Education, Huaqiao University, Quanzhou, China; ^3^GanZhou No1. High School, Ganzhou, China; ^4^School of Education Science, Shangrao Normal University, Shangrao, China; ^5^School of Humanities Education, Yancheng Preschool Teachers College, Yancheng, China

**Keywords:** vertical jumping, Chinese children, learning, development patterns, health promotion

## Abstract

Vertical jumping is the most important of the fundamental motor skills and has an important impact on children's health. A systematic study was conducted on the influence of vertical jumping on children's health and motor development patterns. In this study, the training and learning of vertical jumping were found to be effective in promoting children's development, especially in terms of height and muscle growth. Training interventions were used to determine the influence of age on children's learning of the correct vertical jumping motor pattern. Sex was found to have no influence on children's learning of vertical jumping. Although children were found to be able to learn the correct vertical jumping motor pattern, they could not reach a level of proficiency and intuitively apply the acquired skills to task completion. Cognitive ability was found to have a crucial effect on motor learning among children, especially when they faced various task constraints.

## Introduction

Childhood is one of the most crucial periods of human development, particularly for motor development, including the development of gross motor skills. However, the Chinese government only started focusing on physical exercise for children in the 21st century, which is almost 50 years later than the developed countries in the West. The Chinese government clearly proposed children's recreational rights and physical education rights in the *Outline for the Development of Chinese Children* in 2001 and rapidly began to promulgate and implement related policies. The questions related to children's participation in physical education in the *Advice of Strengthening Teenagers' Physical Education and Constitution* promulgated in 2007 are listed in the “Reference Implementation” document. In the *State Council's Notification of the National Fitness Program (2016–2020), The “Thirteenth Five-Year” Plan of Teenagers' Physical Education*, and “*Healthy China 2030” Program and Outlines* promulgated in 2016, the importance and key points of implementation of children's physical education are clearly stated. In addition, physical education is considered a fundamental public physical education service for children. In the 2019 *Development Notification of Kindergarten Trials Featuring Soccer Coaching of the General Office of the Ministry of Education*, children's soccer was included for the first time in a school soccer development strategy at a national level. The promulgation and implementation of such policies effectively promoted the development of children's physical education in preschools in China. Children's physical education development is expected to accelerate in China in the future.

Due to the fact that children's physical education was focused on at a later stage in China, the development of public participation, preschool implementation, parental awareness, and related scientific research differs considerably from that in developed countries. Consequently, research in this field should reference the successful experiences of developed countries to promote awareness regarding children's physical education at the preschool age. Foreign scholars and childcare workers have extensively studied the development of fundamental motor skills (FMS) in preschool children (1–3). Gallahue and Donnelly indicated that age 3–7 is the golden age to acquire FMS (1). After this age, children encounter difficulty in developing FMS and specific sport skills. Therefore, a motor skill perspective suggests that skill-based activities/games, with instructions, should be encouraged during school-based physical activity and health promotion programs in childhood education (1, 4). The jumping motor action is considered one of the most important motor actions that demonstrate FMS, and it is also one of the first motor actions that children must learn and master.

There has been no systematic research on the formation, learning, and development of the vertical jumping motor action^1^ in preschool Chinese children. In this systematic research, the patterns and characteristics of the formation of the correct jumping motor pattern in Chinese children were analyzed to determine their learning features. The study results will provide guidance to child physical education workers on the instruction of the correct jumping form and will be relevant for child development researchers studying children's overall development and the development of lower extremity motor skills. Thus, this study lays the foundation for child physical education researchers to gain a comprehensive understanding of the patterns and characteristics of children's jumping motor skill development in further research.

## Theoretical Foundation

This study is mainly based on human motor development theory and FMS theory, which are the key theories in research on human development, school physical education, child physical education, and competitive physical education in developed Western countries.

### Human Motor Development Theory

The human motor development of individuals begins in the womb during late pregnancy and progresses until the end of life. Motor development is essential for daily-life activities, including studying, working, and exercising, and has been extensively studied in the physical education and developmental psychology fields. Infancy is characterized by a dramatic increase in motor abilities: the infant learns to reach and grasp; to sit, stand, and walk; and to chew and talk. Initially, these developmental changes were thought to be caused by an evolution from infantile reflexes to cortically controlled behavior (1, 3). However, during the last four decades, two aspects became clear: motor behavior is not primarily organized in terms of reflexes; and already at fetal age, the cortex is involved in modulating motor behavior (4). Human motor development theory can be traced back to the book, *Diary on the Recording of the Infant*. The book records the changes in perception, motor skills, and language acquisition in his two-and-a-half-year-old son. Subsequently, Darwin studied human motor development by recording his children's developmental changes (5). Fischer and Hencke extended the research ideas and demonstrated the interactive relationship between motor development and human perception development, thus establishing the foundation of child psychology and genetic epistemology (6). Moreover, Piaget pointed out that human knowledge originates from human motor skills.

Human motor development has been studied in the fields of psychology, anthropology, biology, medicine, and physical education. Based on research extending over 100 years, Payne and Isaacs concluded that motor development affects humans in multiple aspects (7). First, motor development has a positive influence on human development in terms of intelligence, emotions, and social adaptability. Second, the study of motor development in normal people can be applied to the diagnosis of abnormal developmental processes. Third, people can be trained in physical education by employing scientific methods. Additionally, from the perspective of sport, motor development promoted through motor skills learning in childhood has a positive influence on sport skills learning and participation in physical activities through adolescence, adulthood, and older adulthood (8, 9).

### Fundamental Motor Skills

Fundamental motor skills are a set of gross motor skills that involve the participation of different parts of the body such as the legs, torso, arms, and head in performing various movements. This is considered the prerequisite model for professional sports such as competitive sports, sports games and competitions, and outdoor education, as well as different types of high-skill sports (1). Peiper first proposed the terminology FMS when studying the effects of rhythmic accompaniment on fundamental skills learning in children (3). He proposed that FMS included the motor skills of throwing, catching, climbing, balancing, jumping, bouncing a ball, and striking. Spodek and Saracho analyzed FMS in greater depth by reviewing previous studies (10). They categorized FMS into two types: fundamental locomotor skills and fundamental manipulative skills. This classification was recognized and has been consistently used by researchers in subsequent research (11–13). Balance and stability skills are crucial in child development and sports training practice. In 2002, Gallahue and Ozmun studied motion development in different populations and categorized FMS into three types, namely, locomotor skills, object-control skills, and stability skills (14).

In the early years, when cognition is underdeveloped, there is likely to be a weak correlation between perceived and actual motor competence. This is valuable because children may persist in activities in which they perceive themselves to be competent, which can drive skill acquisition. However, in later childhood, when children are more cognitively developed, the correlation between perceived and actual motor competence is stronger. Children with low motor competence will have low perceived motor competence and engage in less physical activity. This, in turn, might increase the risk of developing conditions associated with physical inactivity such as obesity, which would ultimately provide negative feedback concerning children's motor competence and subsequent physical activity levels. By examining what mediates the relationship between physical activity and FMS, we can determine the underlying mechanism by which one influences the other and highlight potential targets for intervention (1, 3, 5, 15).

At present, FMS are considered one of the major goals of preschool and elementary school physical education in developed countries in Europe, America, and Hong Kong. They are also fundamental in child physical education in these countries and regions (10, 15). Some research studies have stated that preschool is the golden age for FMS development (15–17). After this age, children encounter difficulty in learning FMS and sports-specific skills (17–20). Chang et al. further indicated that FMS are equivalent to fundamental knowledge in physical activities (21). Children should have developed sufficiently diverse motor skills between the ages of 1 and 7 years. The development of FMS improves children's full-body exercise coordination abilities and facilitates the learning of complicated sports-specific skills. Further, FMS development can lay a foundation for children to flexibly adapt to various sports environments in the future. Vertical jumping is the most important motor action in FMS and is included in the category of locomotor skills. Vertical jumping improves children's leg strength and their hand and feet coordination and lays the foundation for the development of other FMS.

Based on human motor development theory and FMS theory, the preschool age is a critical period for motor learning and neural development. Gross muscular training and fundamental motor learning are the major methods used to promote growth and intellectual development in children. Beyond this age, children may have difficulty in learning the correct FMS, which may further cause difficulty in learning sports-specific skills and sports participation during school and adulthood.

## Participants and Methodology

### Participants

This study analyzed the formation process and learning characteristics of the correct jumping motor pattern in kindergarten children in China. A total of 196 children aged 3–6 years and 47 adults (>18 years old) who had received professional training in the jumping motor action were selected as the study participants. Among them, 96 children and 47 adults formed the control group, while 100 children formed the experimental group. The control group (children) comprised 47 boys and 49 girls (average age: 4.6 years), while the control group (adults) comprised 37 men and 10 women (average age: 19.5 years). Among the 100 children in the experimental group, 10 children were excluded due to various reasons (e.g., medical condition, child consent, and family reasons). The experimental group comprised 69 boys and 21 girls (average age: 4.5 years).

All participants were from Quanzhou in Fujian Province, China. The children were enrolled at five preschools in Quanzhou, while all the adult jump experts were based at the School of Physical Education in Huaqiao University. All of the adults had more than 5 years of experience in jumping training.

### Methodology

This study used an experimental design to administer an FMS training intervention to participants. The changes before and after the training intervention and the differences with and without intervention were compared. In addition, the differences between children and skilled people (novices and experts) were compared after the intervention.

The experiment aims (1) to longitudinally compare the changes in the jumping motor action before and after the training intervention among children; (2) to cross-sectionally compare the differences in the jumping motor action between children who received the FMS training intervention and children who underwent regular kindergarten activity courses; and (3) to compare motor differences between children who received the training intervention and skilled people under various task constraints.

#### Experimental Design

(1) *Pretest*: To ensure the normal progress of the training intervention, all the participants underwent pretraining for 1 week. The training comprised simple physical activities and games, which enabled children to rapidly adapt to the formal training environment and stimulated their interest in participation. In the pretest, a detailed explanation of the study purpose and experiment was provided to the parents of children participating in the training; following this, they provided consent to allow their children to participate in the experiment. (2) *Formal training intervention*: With reference to FMS theory and related courses in America and Australia, the study course was designed with a primary focus on vertical jumping and a secondary focus on running and throwing. The course comprised 24 lessons with training activities over a period of 3 months. Each lesson was 1 h long with two lessons a week and eight lessons a month. The coaches who participated in the training intervention were graduate students in physical education who had expertise in child FMS theory and had 1 year of teaching experience. To ensure intervention consistency, all eight graduate students who participated in the training underwent professional training for 1 week before the experiment.

#### Assessment Tool

In this study, children's jumping motor action was assessed using the jumping motor assessment scale in the FMS training instruction book, *Fundamental Motor Skills: Learning, Teaching, and Assessment*, published by the Education Department of Western Australia. The assessment scale assesses the jumping motor action in terms of three stages, namely, preparation stage, hanging stage, and landing stage (Table 1) (18, 21, 22).

**Table 1:** Assessment scale of the jump test.

**Stage**	**Preparation**			**Take off**			**Landing**	
**Part**	**Legs**	**Head and torso**	**Arms**	**Legs**		**Arms**	**Legs**	
Standards	Bent ankles, knees, and hip	Eyes looking forward	Swinging behind the body	Straightening	Leaving the ground simultaneously	Swinging forward and upward	Landing simultaneously	buffer
Score	3	3	3	3	3	3	3	3

*Source: Fundamental motor skills: learning, teaching, and assessment*.

#### Jump Performance Assessment

To ensure an accurate rating of participants' jump performance, this study adopted a double-blind assessment method. Before the official rating, the two assessors (physical education graduate students who did not participate in the training intervention) underwent jump assessment training and qualification testing for 2 days. After passing the qualification test, the two assessors rated the jumping motor recordings (including front-view and side-view recordings) of every child and expert included in the study. A consistency score of 0.77^**^ was obtained (*P* < 0.01).

## Results

### Effect of Jumping Training on Children's Growth and Development

#### Effect on Children's Height and Weight

The height and weight of participants in the experimental group were measured before and after the training intervention. The height of the children after the intervention was significantly higher than before the intervention (*t* = 2.03, *P* = 0.04 < 0.05), yet no significant difference was noted in the weight of the children before and after the intervention (*t* = 0.74, *P* = 0.46 > 0.05). Moreover, no significant difference was observed in the children's muscle mass before and after the intervention (*t* = 1.17, *P* = 0.24 > 0.05). A cross-sectional comparison of the children's height revealed significantly larger height values for the children in the experimental group than for the children in the control group (*P* = 0.05). However, the children in the experimental group exhibited significantly lower weight than those in the control group (*P* = 0.00 < 0.01).

To avoid interference caused by natural growth in height among the participating children, a one-sample *t*-test was conducted to evaluate the height increase in the children participating in the training intervention, with the average height increase in Chinese children (4.5 years old) serving as the reference index. The results revealed that the height increase in the participating children was significantly larger than the average height increase in children in China (boys: *t* = 2.98, *P* = 0.00 < 0.01; girls: *t* = 2.59, *P* = 0.01 < 0.05).

No significant differences in weight and muscle mass were observed in the children before and after training. However, a correlation analysis of the change in weight (average increase: 0.32 kg) and muscle mass (average increase: 0.35 kg) revealed a significant correlation between the two (*P* = 0.00 < 0.01). This indicated that after learning FMS, the change in children's weight was mainly caused by a change in muscle mass.

#### Correlations Between Jumping Training and Children's Body Mass Index

To examine the effect of jumping training on children's BMI, the pretest and post-test results of all the children participating in the experiment were rated according to large-sample BMI test results provided in previous studies (1, 2). Children in the normal interval were given a score of 3 [BMI: 15.4 ± 1.2 (age 3), 15.3 ± 1.3 (age 4 and 5), and 15.4 ± 1.4 (age 6)]; those in the slightly underweight or slightly overweight interval were given a score of 2 [BMI: 13.2–14.3 or 16.8–18.2 (age 3), 13–14 or 16.6–18.2 (age 4 and 5), and 13.1–14.1 or 16.9–18.7 (6 years)]; and those in the severely underweight or obese overweight were given a score of 1 [BMI: <13.2 or >18.2 (age 3), <13 or >18.2 (age 4 and 5), and <13.1 or >18.7 (age 6)]. An independent sample *t*-test was conducted on the scores of the pretest and post-test, which revealed no significant difference between the two test results (*t* = 1.75, *P* = 0.08 > 0.05). However, more than 50% of the extremely underweight and overweight children exhibited favorable changes in their body shape after training.

#### Jumping Motor Performance of the Experimental and Control Groups at Different Stages

Table 2 shows that there were significant differences between the experimental and control groups in children's total scores for the jumping motor training (*P* = 0.000 < 0.01). The total score for the motor development sequence of the children in the experimental group was significantly higher than that of those in the control group. The children in the two groups exhibited significant differences in the preparation and takeoff stages (*P* = 0.00 < 0.01), but not in the landing stage (*P* > 0.05).

**Table 2:** Total score comparison at different stages of jumping motor between the groups.

	**Experimental group**		**Control group**		***t***	***P***
	**Mean**	**Standard**	**Mean**	**Standard**		
		**deviation**		**deviation**		
Total score	37.13	9.84	23.17	7.74	10.78^**^	0.00
Preparation stage	7.31	1.59	4.81	0.92	13.21^**^	0.00
Takeoff stage	6.64	2.52	5.07	1.35	5.35^**^	0.00
Landing stage	4.57	1.74	4.27	1.16	1.37	0.17

^*^P < 0.05,

** *P < 0.01*.

#### Upper and Lower Limb Motor Performance of the Experimental and Control Groups at Different Stages

The ANOVA on scores for all the three jumping stages (i.e., the preparation stage, takeoff stage, and landing stage) of the children in the experimental group revealed no significant differences (*F* = 2.01, *P* = 0.136 > 0.05). In the control group, however, the scores in the landing stage were significantly higher than those in the preparation and takeoff stages (*F* = 4.34, *P* = 0.00 < 0.01).

An analysis of the two groups (Table 3) revealed that the control group exhibited significant differences in upper and lower limb motor performance after the training intervention (*P* = 0.00 < 0.01). However, in the experimental group, no significant difference was noted after the intervention (*P* = 0.281 > 0.05).

**Table 3:** Comparison of the upper limb and lower limb motors between the experimental and control groups.

	**Upper limb motor**		**Lower limb motor**		***t***	***P***
	**Mean**	**Standard deviation**	**Mean**	**Standard deviation**		
Experimental group	2.38	0.94	2.23	0.91	1.08	0.28
Control group	0.36	0.45	2.33	0.61	−25.44^**^	0.00

*P < 0.05,

** *P < 0.01*.

### Effects of Age and Sex on the Formation of Children's Jumping Motor Skills

#### Effect of Age on the Jumping Motor Performance of Children Before the Training Intervention

The ANOVA on the post-training scores for the jumping motor performance of children aged 3–4 years, 4–5 years, and 5–6 years revealed significant differences at different ages (*F* = 3.82, *P* = 0.03 < 0.05).

To determine the stage of the jumping motor action that accounts for the differences in jump performance between children of different ages, a *post-hoc* analysis of the three jumping-stage scores was conducted. The results indicated significant differences mainly in the preparation and takeoff stages but not in the landing stage in children of different ages (Table 4).

**Table 4:** Comparison of the three-stage jump performance in children of different ages before the training intervention.

**Stage**	**Age**	***n***	**Mean**	**Standard**	**Mean**	***P***
	**(years)**			**deviation**	**deviation**	
Preparation	3-4	30	4.20	1.13	−0.92^**^	0.00
	4-5	33	5.12	0.74		
	3-4	30	4.20	1.13	−0.86^**^	0.00
	5-6	33	5.06	0.56		
	4-5	33	5.12	0.74	0.06	0.96
	5-6	33	5.06	0.56		
Takeoff	3-4	30	4.80	1.47	0.13	0.92
	4-5	33	4.67	1.14		
	3-4	30	4.80	1.47	−0.93^*^	0.02
	5-6	33	5.73	1.21		
	4-5	33	4.67	1.14	−1.06^**^	0.01
	5-6	33	5.73	1.21		
Landing	3-4	30	4.17	1.49	−0.17	0.85
	4-5	33	4.33	4.33		
	3-4	30	4.17	1.49	−0.14	0.89
	5-6	33	4.30	1.08		
	4-5	33	4.33	4.33	0.03	0.99
	5-6	33	4.30	1.08		

* *P < 0.05*,

** *P < 0.01*.

#### Effect of the Training Intervention on the Jumping Motor Performance of Children of Different Ages

To evaluate the difference in the jumping motor performance between children of different ages after the training intervention, an ANOVA was conducted. The results indicated no significant difference in the overall jumping performance among children of different ages after the training intervention. Further analysis showed no significant difference in the jumping motor performance at the preparation, takeoff, and landing stages (Table 5). This finding indicated that the training intervention could improve children's jumping motor performance, mainly in the preparation and takeoff stages. Moreover, the training intervention allowed children aged 3–6 years to achieve the same jumping motor performance.

**Table 5:** Effect of the training intervention on the jumping motor performance among children of different ages.

	**Age**	***N***	**Mean**	**Standard**	**Mean**	***P***
	**(years)**			**deviation**	**deviation**	
Overall	3-4	13	17.31	6.54	−1.49	0.67
	4-5	29	18.79	5.41		
	3-4	13	17.31	6.54	−1.38	0.67
	5-6	48	18.69	4.14		
	4-5	29	18.79	5.41	0.11	1.00
	5-6	48	18.69	4.14		
Preparation	3-4	13	6.92	1.98	−0.46	0.70
	4-5	29	7.38	1.70		
	3-4	13	6.92	1.98	−0.45	0.67
	5-6	48	7.37	1.42		
	4-5	29	7.38	1.70	0.00	1.00
	5-6	48	7.37	1.42		
Takeoff	3-4	13	5.85	3.13	−0.95	0.53
	4-5	29	6.79	2.64		
	3-4	13	5.85	3.13	−0.92	0.51
	5-6	48	6.77	2.27		
	4-5	29	6.79	2.64	0.02	1.00
	5-6	48	6.77	2.27		
Landing	3-4	13	4.54	1.94	−0.08	0.99
	4-5	29	4.62	1.82		
	3-4	13	4.54	1.94	0.00	1.00
	5-6	48	4.54	1.68		
	4-5	29	4.62	1.82	0.08	0.98
	5-6	48	4.54	1.68		

#### Effect of Sex on the Jumping Motor Performance of Children

A comparison of the scores for jumping motor performance by sex revealed no significant difference in the jumping motor skills of boys and girls in the control group. The jumping performance did not differ between boys and girls of the same age without training intervention. Moreover, no significant difference in the jumping motor performance was observed between boys and girls in the experimental group. After the training intervention, the boys and girls of the same age exhibited no differences in jumping performance (Table 6). This finding indicates that sex had no effect on the jumping motor performance. Therefore, similar training intervention standards can be used for boys and girls.

**Table 6:** Effect of sex on children's jumping motor performance.

**Group**	**Age**	**Sex**	***N***	**Mean**	**Standard**	***P***
	**(years)**				**deviation**	
Control group	3-4	Female	18	13.61	2.68	0.42
		Male	12	12.50	4.70	
	4-5	Female	17	14.24	2.44	0.77
		Male	16	14.00	2.03	
	5-6	Female	14	15.50	1.40	0.40
		Male	19	14.79	2.86	
Experimental group	3-4	Female	7	19.00	4.43	0.33
		Male	6	15.33	8.38	
	4-5	Female	5	22.60	1.67	0.08
		Male	24	18.00	5.60	
	5-6	Female	9	17.33	5.32	0.28
		Male	39	19.00	3.83	

### Effect of Different Task Constraints on Jumping Motor Performance

#### Children's Jumping Motor Performance in Tasks With Different Difficulty Levels

A multivariate ANOVA was conducted to compare the overall jumping motor performance after completion of tasks with three difficulty levels among children aged 3–6 years. The results revealed significant differences in the overall jumping motor performance of children during tasks with different difficulty levels (*P* = 0.00 < 0.01). A further *post-hoc* analysis indicated large differences in the mean scores of children between high-difficulty tasks and medium- and low-difficulty tasks, with the differences mainly being in the scores for high-difficulty tasks (Table 7).

**Table 7:** *Post-hoc* analysis of the overall scores of children after completion of tasks with varying difficulties.

**Group**		**Mean difference**	**Standard error**	***P***
Low difficulty	Medium difficulty	1.78	0.77	0.73
	High difficulty	7.13^**^	0.77	0.00
Medium difficulty	Low difficulty	−1.78	0.77	0.73
	High difficulty	5.36^**^	0.77	0.00
High difficulty	Low difficulty	−7.13^**^	0.77	0.00
	Medium difficulty	−5.36^**^	0.77	0.00

^*^P < 0.05,

** *P < 0.01*.

To further evaluate the jumping performance of children at different motor stages after completion of tasks with different difficulty levels, a multivariate ANOVA was conducted. The results revealed that in completing tasks with different difficulty levels, no significant difference was observed in the performance at the preparation stage (*F* = 0.30, *P* = 0.74 > 0.05), but extremely significant differences were noted in the takeoff (*F* = 41.76, *P* = 0.00 < 0.01) and landing stages (*F* = 74.87, *P* = 0.00 < 0.01). A further *post-hoc* analysis revealed that the children generally had lower scores in the jumping and landing stages when completing high-difficulty tasks. The mean differences between the high-difficulty scores and medium- and low-difficulty scores were large (*P* = 0.00 < 0.01, *P* = 0.00 < 0.01). In the takeoff stage, significant differences in the children's degree of motor completion were noted for the medium- and low-difficulty tasks (*P* = 0.01 < 0.05; Table 8).

**Table 8:** Variance analysis *post-hoc* comparison of children's performance in tasks with different difficulty levels at different stages.

**Stage**	**Group**		**Mean**	**Standard**	***P***
			**difference**	**error**	
Takeoff	Low difficulty	Medium difficulty	0.38^*^	0.13	0.01
		High difficulty	1.14^**^	0.13	0.00
	Medium difficulty	Low difficulty	−0.38^*^	0.13	0.01
		High difficulty	0.77^**^	0.13	0.00
	High difficulty	Low difficulty	−1.14^**^	0.13	0.00
		Medium difficulty	−0.77^**^	0.13	0.00
Landing	Low difficulty	Medium difficulty	0.21	0.16	0.43
		High difficulty	1.83^**^	0.16	0.00
	Medium difficulty	Low difficulty	−0.21	0.16	0.43
		High difficulty	1.62^**^	0.16	0.00
	High difficulty	Low difficulty	−1.83^**^	0.16	0.00
		Medium difficulty	−1.62^**^	0.16	0.00

* *P < 0.05*,

** *P < 0.01*.

#### Experts' Jumping Motor Performance in Tasks With Different Difficulty Levels

A multivariate ANOVA was conducted to evaluate experts' overall step jumping motor performance in the tasks with three difficulty levels. The results showed that the differences in the overall jumping motor performance of experts were extremely significant in task constraints with different difficulty levels (*P* = 0.00 < 0.01). The *post-hoc* comparison of the three different difficulties showed that experts' motor performance scores exhibited large mean differences for all three levels of difficulty (*P* = 0.00 < 0.01). Moreover, significant differences in the experts' degree of motor completion were noted in tasks with different difficulty levels.

Experts' jumping motor skills under different levels of difficulty were compared at different stages. The results indicated extremely significant differences in completion of tasks with different levels of difficulty at the preparation (*F* = 80.67, *P* = 0.00 < 0.01), takeoff (*F* = 140.41, *P* = 0.00 < 0.01), and landing stages (*F* = 26.19, *P* = 0.00 < 0.01). A *post-hoc* comparison of the variance analysis results of the experts' performance at the three stages revealed that significant differences were mostly noted in the scores of the preparation and takeoff stages (*P* = 0.00 < 0.01; Table 9). In the landing stage, experts exhibited higher scores for high-difficulty tasks than for tasks with the other two difficulty levels (*P* = 0.00 < 0.01; Table 9).

**Table 9:** *Post-hoc* comparison of variance analysis of experts' performance at different stages in tasks with different difficulty levels.

**Stage**	**Group**		**Mean**	**Standard**	***P***
			**difference**	**error**	
Preparation	Low difficulty	Medium difficulty	−0.57^**^	0.08	0.00
		High difficulty	−1.03^**^	0.08	0.00
	Medium difficulty	Low difficulty	0.57^**^	0.08	0.00
		High difficulty	−0.46^**^	0.08	0.00
	High difficulty	Low difficulty	1.03^**^	0.08	0.00
		Medium difficulty	0.46^**^	0.08	0.00
Takeoff	Low difficulty	Medium difficulty	−0.30^**^	0.07	0.00
		High difficulty	−1.14^**^	0.07	0.00
	Medium difficulty	Low difficulty	0.30^**^	0.07	0.00
		High difficulty	−0.85^**^	0.07	0.00
	High difficulty	Low difficulty	1.14^**^	0.07	0.00
		Medium difficulty	0.85^**^	0.07	0.00
Landing	Low difficulty	Medium difficulty	−0.19	0.09	0.10
		High difficulty	−0.67^**^	0.09	0.00
	Medium difficulty	Low difficulty	0.19	0.09	0.10
		High difficulty	−0.48^**^	0.10	0.00
	High difficulty	Low difficulty	0.67^**^	0.09	0.00
		Medium difficulty	0.48^**^	0.10	0.00

^*^P < 0.05,

** *P < 0.01*.

#### Experts' and Children's Jumping Motor Performance in Tasks With Different Difficulty Levels

The overall performance of experts and children in the completion of tasks with different levels of difficulty was compared. The results indicated that both experts and children exhibited extremely large differences in the overall scores in low-difficulty (*t* = 12.42, *P* = 0.00 < 0.01), medium-difficulty (*t* = 19.70, *P* = 0.00 < 0.010), and high-difficulty (*t* = 51.49, *P* = 0.00 < 0.01) tasks. A detailed comparison of the scores between the two groups at each jumping stage for tasks of different levels of difficulty revealed extremely significant differences in the preparation and takeoff stages in low-difficulty and high-difficulty tasks (*P* = 0.00 < 0.01; Table 10). Moreover, significant differences were noted in the landing stage in medium-difficulty and high-difficulty tasks (*P* = 0.03 < 0.05; *P* = 0.00 < 0.01).

**Table 10:** Comparison of jumping motor performance between experts and children at different stages in tasks with different difficulties.

			***N***	**Mean**	**Standard**	***t***	***P***
					**deviation**		
Low difficulty	Preparation	Experts	47	1.94	0.51	−4.53^**^	0.00
		Children	89	2.43	0.65		
	Takeoff	Experts	47	1.83	0.43	−4.26^**^	0.00
		Children	89	2.40	0.87		
	Landing	Experts	47	2.33	0.64	0.16	0.87
		Children	89	2.30	1.02		
Medium difficulty	Preparation	Experts	45	2.37	0.43	0.08	0.94
		Children	89	2.36	0.80		
	Takeoff	Experts	45	2.13	0.29	0.69	0.49
		Children	89	2.03	0.93		
	Landing	Experts	45	2.52	0.32	2.26^*^	0.03
		Children	89	2.09	1.26		
High difficulty	Preparation	Experts	37	2.96	0.10	7.13^**^	0.00
		Children	89	2.42	0.46		
	Takeoff	Experts	37	2.97	0.09	13.99^**^	0.00
		Children	89	1.26	0.74		
	Landing	Experts	37	3.00	0.00	15.69^**^	0.00
		Children	89	0.47	0.98		

* *P < 0.05*,

** *P < 0.01*.

## Discussion

### The Proposed Jumping Motor Training Intervention Could Effectively Facilitate Children's Growth and Development

The results showed that children who participated in FMS training had significant differences in height before and after training compared with those in the control group. In addition, the height increases in the children who participated in the training were significantly higher than the average increase in children of the same age in China. These results are consistent with previous studies which reported that FMS training, particularly jumping motor training, can significantly increase the bone mass of the lower limbs and spine, increase bone density, and facilitate height increase in children (8, 19–21). Regarding weight, no significant differences were seen before and after training. Regarding muscle mass, children in the experimental group exhibited a significant increase. These results indicate that the children who participated in the training exhibited favorable changes in their body shapes. Longer and continual training could result in significantly favorable changes in children's growth and development.

Body shape also influences the learning of FMS, including the jumping motor action. Previous studies indicated that proficiency in FMS is significantly correlated with children's body shape (19, 22, 23). The FMS of children with normal weight are two to three times higher than that of overweight children (3, 22). This study also found that the jumping motor performance of overweight and underweight children was poorer than that of children with a normal body shape. This may be attributed to the problem of insufficient leg strength influenced by body shape.

### Development Sequence of the Jumping Motor Action in Chinese Children Differed From That of Children in Other Countries

Evidence from international research has indicated that the development of FMS has certain stage characteristics, termed the motor development sequence. From the perspective of the total body approach to motor development, most children are expected to exhibit more consistent development sequence characteristics in the process of motor learning (3, 23, 24). In this study, Chinese children exhibited the following characteristics in the development of the jumping motor action: the correct jumping motor pattern was formed through 3 months of training intervention; the motor action at the landing stage was formed first (even without training); and the motor action at the preparation and takeoff stages was formed after the training intervention. However, at which of the two stages the motor action was first formed could not be concluded. The conclusions of this study differ from those reported in children in the West (2, 24) in terms of learning time and the learning sequence of motor actions. In terms of learning time, most 3–6-year-old Chinese children can grasp the jumping motor action within 3 months of the training intervention. However, foreign scholars believe that most children can only learn the jumping motor action at around 10 years of age. Regarding the learning sequence of motor actions, this study verified that the landing buffer motor action was the first to develop in Chinese children. However, foreign scholars believe that the arm motor action for maintaining balance and forward swing of the arms is developed first. Both domestic and foreign studies have indicated that the development of upper limb motor actions is relatively slow in the learning process of jumping. The jumping motor training particularly focused on the upper limbs.

### Age Influenced Jumping Motor Skill Development in Children, but Not Jumping Motor Learning

This study discovered that children of different ages exhibited different jumping motor characteristics without intervention. After the intervention, children of different ages all displayed improvement in jump performance. Thus, both the training intervention and increasing age influenced the formation of the correct jumping motor action in children.

In the control group, the performance of older children in the preparation and hanging stages was significantly better than that of younger children. In the preparation stage, the motor differences between children aged 4–6 years and those aged 3–6 years were most noticeable in terms of the ankle, knee, and hip flexion angles. This is consistent with the results observed by Chang et al. (21). According to Hadders-Algra (4), the motor differences are not caused by physiological development problems but cognitive differences. In the takeoff stage, children aged 5–6 years had significantly higher scores than those aged 3–5 years. The differences were mainly in terms of the degree of stretching of the torso and legs, which may be attributed to the increase in leg strength with increasing age. This is because greater height from the ground allows children to more precisely complete the motor action. Further, in the experimental group, children of different ages exhibited no significant difference in performance at different stages of the jump after the training intervention. This result indicated that children of different ages can achieve the same jumping motor performance after training intervention. The conclusions of this study are consistent with the viewpoint of past research (15–19), where FMS were found to be related to but not dependent on age. Moreover, there is no specific development sequence linked to the learning of FMS.

In summary, this study answers two questions. First, the correct jumping motor action is not naturally formed with growth and development. This study compared the jumping performance of children of different ages. However, it was not found that children in the oldest age group had already developed the jumping motor model. Second, the core view of motor development sequence theory is that the development of FMS has stage and temporal characteristics and that there is no concrete formation time and sequence of the motor skills.

### Sex Did Not Influence the Formation and Development of Children's Jumping Motor Skill

Sex has no influence on the development of children's jumping motor skills. We found no significant difference in jumping performance between boys and girls of the same age in the experimental and control groups after the intervention. This result indicates that jumping motor skill development and learning in preschool children are not associated with sex. Eccles et al. indicated that girls who live in families with gender stereotypes (e.g., where boys are considered more athletic than girls) will have poorer sports performance in the future than those who live in families without gender stereotypes (15, 22). They demonstrated that numerous people do believe that men are naturally more athletic than women (22, 25). Their study also indicated that girls' perception of their own athleticism being high or low is often influenced by parents' expectations and family awareness. Nevertheless, this study proved that preschool girls and boys do not exhibit significant differences in jumping motor performance.

### Children's Jumping Motor Development Did Not Reach the Stage of Proficiency

This study verified that the children in the experimental group established the correct jumping motor model after 3 months of training. However, when they performed the jumping motor action (in completing tasks), their jumping motor patterns changed. This verified the viewpoint of Potter et al. (23), who stated that children's motor development is influenced by individuals, the environment, and tasks. After children learn the correct jumping pattern, they may still not be able to perform the correct motor action to complete the jump in related tasks at any time.

This study used the novice–expert model to evaluate the jumping motor patterns of children and adults who received professional training when they completed tasks with varying difficulty levels. Children's accuracy in the jumping motor action decreased with increasing difficulty. By contrast, experts' motor accuracy increased. These results may be attributed to cognitive and decision-making abilities. Experts have a more complete decision-making cognitive structure and, as a result, demonstrate better information selection and decision-making skills (5–7). They knew that the correct jumping motor pattern is beneficial for completing more difficult tasks. These results verified Piaget's theory, which proved that cognitive development has a certain influence on the generation, execution, and results of motor actions (12, 25, 26).

### Rational Use of Task Constraints to Promote Jumping Teaching

This study found that children's accuracy in the jumping motor action decreased with increasing task difficulty. In the teaching process of children's vertical jumping, we should not excessively increase the jump practice height, but pay more attention to the process of children's action completion. Moreover, we should focus on the correctness of the jump motor action, not height, when we evaluate children's learning. Therefore, when we design a children's jumping class, we should consider the level of every child and set a reasonable height as a task constraint to make sure all children complete the task.

## Limitations and Future Directions

Children learned the correct jumping motor pattern after training intervention. However, due to the cognitive difference and shorter training time, they could not intuitively apply their acquired motor skills in practice. Therefore, children's physical education teachers must understand that motor learning is a long-term process. The correct fundamental motor pattern must be taught to children through images. In addition, children must be allowed to have deeper and more comprehensive knowledge of the correct motor action through verbal instructions and task design.

Future research directions include focusing on another motor skill involved in FMS development and teaching study, studying the factors of influence in the jump development of children, and the popularization of FMS in preschools in China.

## Data Availability Statement

The raw data supporting the conclusions of this article will be made available by the authors, without undue reservation.

## Ethics Statement

The studies involving human participants were reviewed and approved by the Ethical Committee of the Sichuan University. Written informed consent to participate in the study was provided by the participants' legal guardian/next of kin.

## Author Contributions

ZZ, QP, MC, and LL contributed to the inception, design, and data collection of the teaching experiment. Data analysis was performed by LL and WY. The writing and editing of the manuscript were performed by ZZ, QP, MC, LX, and LL. All authors contributed to the article and approved the submitted version.

## Funding

This research was supported by the Fundamental Research Funds for the Central Universities in Sichuan University and Sichuan University (2021CXC26).

## Conflict of Interest

The authors declare that the research was conducted in the absence of any commercial or financial relationships that could be construed as a potential conflict of interest.

## Publisher's Note

All claims expressed in this article are solely those of the authors and do not necessarily represent those of their affiliated organizations, or those of the publisher, the editors and the reviewers. Any product that may be evaluated in this article, or claim that may be made by its manufacturer, is not guaranteed or endorsed by the publisher.

## Footnotes

^1^Hereafter, “vertical jumping” will be abbreviated to “Jumping”.
